# Arbuscular mycorrhizal fungi communities and promoting the growth of alfalfa in saline ecosystems of northern China

**DOI:** 10.3389/fpls.2024.1438771

**Published:** 2024-08-29

**Authors:** Wen Xu, Qianning Liu, Baiji Wang, Na Zhang, Rui Qiu, Yuying Yuan, Mei Yang, Fengdan Wang, Linlin Mei, Guowen Cui

**Affiliations:** College of Animal Science and Technology, Northeast Agricultural University, Harbin, China

**Keywords:** arbuscular mycorrhizal fungi, alfalfa, salinization, rhizosphere soil, growth-promoting

## Abstract

Arbuscular mycorrhizal fungi (AMF) are universally distributed in soils, including saline soils, and can form mycorrhizal symbiosis with the vast majority of higher plants. This symbiosis can reduce soil salinity and influence plant growth and development by improving nutrient uptake, increasing plant antioxidant enzyme activity, and regulating hormone levels. In this study, rhizosphere soil from eight plants in the Songnen saline–alkaline grassland was used to isolate, characterize, and screen the indigenous advantageous AMF. The promoting effect of AMF on alfalfa (*Medicago sativa* L.) under salt treatment was also investigated. The findings showed that 40 species of AMF in six genera were identified by high-throughput sequencing. *Glomus mosseae* (G.m) and *Glomus etunicatum* (G.e) are the dominant species in saline ecosystems of northern China. Alfalfa inoculated with *Glomus mosseae* and *Glomus etunicatum* under different salt concentrations could be infested and form a symbiotic system. The mycorrhizal colonization rate and mycorrhizal dependence of G.m inoculation were significantly higher than those of G.e inoculation. With increasing salt concentration, inoculation increased alfalfa plant height, fresh weight, chlorophyll content, proline (Pro), soluble sugar (SS), soluble protein (SP), peroxidase (POD), superoxide dismutase (SOD), and catalase (CAT) activity while decreasing the malondialdehyde (MDA) content and superoxide anion production rate. The results highlight that inoculation with G.m and G.e effectively alleviated salinity stress, with G.m inoculation having a significant influence on salt resistance in alfalfa. AMF might play a key role in alfalfa growth and survival under harsh salt conditions.

## 1 Introduction

Soil salinization, resulting from poor irrigation management and drought, is one of the most destructive environmental stresses causing significant declines in arable land area, crop productivity, and quality globally (Shahbaz and Ashraf, 2013). The Songnen Plain, one of the three main grasslands in northeastern China, has approximately 15.24% of its area covered with saline soil. Factors such as natural changes (climate and soil-forming parent material) and human activities (extensive land clearing and imperfect water conservancy facilities) have contributed to the continuous deterioration of environmental conditions in the Songnen grassland (Wang et al., 2009). Serious soil compaction, destruction of the granular structure, high soil pH, and nutrient depletion (Gong et al., 2021) not only limit plant growth and reduce the yields and quality of most crops but also affect the physicochemical properties of soils and the ecological balance (Shrivastava and Kumar, 2015). Methods such as physical improvement, chemical improvement, and hydraulic improvement have been adopted to address these problems (Zhao et al., 2023). However, these methods are time-consuming and labor-intensive, which, to some extent, hinder the restoration of saline–alkaline soils. Rhizosphere biotrophic bacteria such as plant beneficial bacteria (PBB) and arbuscular mycorrhizal fungi (AMF) can assist plants in surviving and growing under adverse conditions and increase their resistance to salinity, extreme temperature, and heavy metal stress, thereby increasing plant yield (Garg and Chandel, 2011). Among them, AMF have become a research focus in recent years due to their ability to increase plant salinity tolerance and promote plant nutrient uptake.

Alfalfa (*Medicago sativa* L.) is a high-quality leguminous fodder with excellent palatability and a developed root system (Ferreira et al., 2015). It is widely cultivated worldwide and holds significant economic value and ecological importance (Mei et al., 2022a). The growth of alfalfa is severely restricted in agricultural areas with high salinity, particularly during the early stages of seed germination and growth. High salinity inhibits or delays germination and the elongation of branches and roots due to osmotic stress (Li et al., 2010). Research indicates that elevated soil salinity levels decrease the content of trace elements (Fe^2+^, Mn^2+^, Cu^2+^, and Zn^2+^) in the roots, stems, and leaves of alfalfa, disrupting ion transport ratios and cation transport selectivity ratios (Bhattarai et al., 2021). Salt stress also hinders alfalfa growth by reducing plant growth rate and water absorption while increasing proline (Pro) content and membrane peroxidation (Choi et al., 2018). Additionally, salt stress indirectly impacts alfalfa growth by influencing plant metabolism, such as the symbiotic nitrogen fixation ability of rhizobia (Farooq et al., 2017). As salt concentration rises, the nitrogenase activity of alfalfa nodules decreases. Combined salt stress can destroy the nitrogenase component structure in root nodules, reducing the nitrogen fixation capability and ultimately decreasing the total nitrogen content.

AMF are endophytic fungi widely distributed in soil, capable of forming mycorrhizal symbiosis with most terrestrial plants (Zhang et al., 2024a). It is recognized as a biotechnological tool to increase plant resistance and restore ecosystems (Cao et al., 2020). Research has shown that AMF symbionts improve plant resistance by strengthening the water absorption capacity, promoting nutrient assimilation, sustaining ion balance, and increasing photosynthesis and the plant hormone levels (Han et al., 2024; Wang et al., 2024b). Under stress conditions, AMF’s extensive mycelial network extends the rhizosphere absorption area, increasing the plant’s absorption capacity (Liu et al., 2016; Kakouridis et al., 2022). AMF application reduced the negative effects of salt stress by increasing the absorption of microelements, regulating the absorption of Na and K in wheat (Huang et al., 2023). Plants inoculated with AMF exhibit higher transpiration rates and leaf water potential under salt conditions, significantly improving relative leaf water content and survival ability (Akhzari et al., 2016). Mycorrhizal seedling leaves display significantly higher activities of superoxide dismutase (SOD), catalase (CAT), and ascorbate peroxidase (APX) compared to non-mycorrhizal seedlings, helping *Elaeagnus angustifolia* seedlings to cope with salinity (Chang et al., 2018). Furthermore, the levels of proline (Pro), soluble protein (SP), and antioxidant enzymes activity are higher in AMF-inoculated plants under high salt stress, indicating that the beneficial effects of mycorrhizal symbiosis are crucial for managing severe salt toxicity (Zong et al., 2023). Additionally, AMF-inoculated plants show stronger net photosynthetic rates, stomatal conductance, and chlorophyll content under salt stress (Chandrasekaran et al., 2019). AMF inoculation promotes the accumulation of alfalfa biomass under salt stress and increases the stomatal constraints of alfalfa leaves, increasing CO_2_ fixation capacity (Shi-Chu et al., 2019).

Soil salinization markedly inhibits plant growth and severely limits agricultural productivity. Therefore, the restoration of saline-alkali land has become an urgent problem to solve. The Songnen saline-alkali grassland, characterized by specific habitats and rich microbial resources, primarily comprises the dominant groups *Ascomycota*, *Basidiomycota*, and *Mortierellomycota* (Cui et al., 2023), making it a vital strategic resource for microbial strains. The unique environment of this grassland may lead to regional specificity in AMF. Alfalfa, an important forage, was selected as the experimental material due to its high yield, rich nutritional value, and strong stress resistance. However, its growth and development are severely limited in saline-alkali soils (Guo et al., 2024). AMF have been shown to significantly alleviate plant salinity stress (Evelin et al., 2009; Qin et al., 2021), and the role of AMF in enhancing salt stress tolerance in alfalfa has received increasing attention. Previous studies have outlined various mechanisms and strategies by which AMF alleviate salinity stress in alfalfa (Moradi, 2016; Laouane et al., 2019; Shi-Chu et al., 2019). However, the effects of the native AMF species in Songnen grassland to alfalfa salinity stress are still uncertain. Most of the strains used in prior studies were sourced commercially (Li et al., 2020; Zong et al., 2023; Jia et al., 2024). In our experiment, local strains from the Songnen grassland were cultured and propagated, and alfalfa was inoculated to cope with salt stress, aiming to overcome the poor adaptability of exogenous sourced strains to Northeast China’s conditions. Accordingly, AMF was isolated, screened, and identified from the rhizosphere of eight dominant saline-tolerant plants in the Songnen saline-alkali grassland, and the growth-promoting effects of two native dominant AMF inoculations on alfalfa were evaluated for the first time through a pot experiment. The aims were (1) to identify the diversity characteristics and dominant species of AMF in the rhizosphere of plants of Songnen saline-alkali grassland in salinized areas and (2) to analyze the efficiency of dominant AMF species isolated from the rhizosphere of plants in Songnen saline-alkali grassland to alleviate salt stress in alfalfa.

## 2 Materials and methods

### 2.1 Study site

This study site is located in Songnen grassland (Zhaodong City, Heilongjiang Province, China; 139–140 masl; 46°2′45″–46°3′12″ N, 125°53′51″–125°54′1″ E; **Supplementary Figure S1**). The region experiences hot and rainy summers and cold and dry winters, with a mean annual precipitation of 569.1 mm. The soil pH is 8.2. The predominant plant community consists of *Leymus chinensis* (Trin.) and *Puccinellia tenuiflora* (Griseb.), along with other companion species. Species diversity has decreased due to salinity stress and grazing.

### 2.2 Sample collection and sequencing

All soil samples used in the experiment were collected in September 2019 in Zhaodong, China, from the rhizosphere soil of eight salinity-tolerant plant species (**Table 1**). In the saline plot, three plants of each species were randomly selected, and impurities such as litter and stones were removed from the soil surface. Soil from the plant rhizosphere at a depth of 5–20 cm was dug up. The roots in the soil were shaken to separate loose soil, which was then mixed into one sample, sealed in a plastic bag, and returned to the laboratory on ice. All the rhizosphere soil samples were divided into two parts. One part was naturally air-dried and sieved (2 mm) for soil analysis, and the rest was stored at -80°C for sequencing.

**Table 1:** Distribution locations of eight sampled plants in Songneng grassland.

Hosting plants	Number	East longitude	North latitude
*Arundinella anomala* Steud.	A	46°2′46″	125°53′51″
*Leymus chinensis*	B	46°2′46″	125°53′53″
*Taraxacum mongolicum*	C	46°3′10″	125°53′59″
*Puccinellia tenuiflora*	D	46°3′9″	125°53′58″
*Artemisia mongolica*	E	46°3′12″	125°54′1″
*Artemisia anethifolia*	F	46°3′12″	125°54′0″
*Clematis hexapetala*	G	46°2′54″	125°53′51″
*Vicia amoena*	H	46°3′8″	125°54′0″

DNA from rhizosphere soil samples of eight plants was extracted using the Power Soil DNA Isolation Kit (Mo Bio Laboratories, Inc., USA) according to the manufacturer’s instructions. Three repetitions were set, and quality and purity were checked using 1% agarose gel electrophoresis. Two pairs of primers (AML1F-AML2R and AMV4-5NF_AMDGR) were selected to amplify partial 16s rRNA gene fragments of AMF by nested polymerase chain reaction (PCR). Amplicon libraries were prepared and identified using the Illumina MiSeq sequencing method at Meijorbio Go. (Shanghai, China). Clean sequences were clustered into operational taxonomic units (OTUs) based on 97% similarity, and the most abundant sequences were selected as representative sequences. Annotation through the Silva and Unite databases was used to categorize soil microbial species. AMF alpha diversity indices including Chao, Sobs, Shannon, and Simpson index were calculated using QIIME (http://qiime.org/scripts/assign_taxonomy.html). Significant differences in alpha diversity were assessed with a non-parametric test in QIIME.

### 2.3 Experimental design and treatments

AMF spores were isolated using wet sieving and sucrose centrifugation (McKenney and Lindsey, 1987). The screened substances were stored in a petri dish at 4°C, and each soil sample was processed three times. Individual spores were placed under a light microscope with a pipette to observe their color, shape, and other superficial characteristics. Spore species were determined based on the *Arbuscular Mycorrhizal Fungal Resources and Germplasm Resources of China*, the International Arbuscular Mycorrhizal Fungi Conservation Center (INVAM), and species descriptions and pictures from published articles. Two dominant AMF species spores, *Glomus mosseae* and *Glomus etunicatum*, selected from the Songnen grassland rhizosphere soil were propagated. The propagation method involved sterilizing seedling trays with 75% alcohol, filling them to the top with sterilized quartz sand culture substrate (sterilized for 120 min at 121°C and 103 kPa), and digging a 6- to 7-cm-deep hole with a sterilized glass rod. Under a stereoscopic microscope, fresh, bright, full spores were collected and placed on the roots of *Trifolium repens* L. seedlings. The seedlings were then immediately transferred into pre-dug holes and compacted with glass rods. The setup was left in the dark for 24 h before being transferred to a greenhouse for 3 months (16-h light/8-h dark photoperiod, 23–26°C). Root colonization was checked after 15–20 days. After 3 months of cultivation, the roots and rhizosphere soil were collected.

Five salt concentration gradients (0, 50, 100, 150, and 200 mmol/L NaCl) were set up (Tani et al., 2018; Shi-Chu et al., 2019), with three replicates for each concentration. Three treatments were applied for each salt concentration: control (non-inoculation), inoculation with *Glomus mosseae* (G.m), and inoculation with *Glomus etunicatum* (G.e). The pot experiment was conducted in a greenhouse using the alfalfa variety “Dongnong No. 1” from Northeast Agricultural University. All the seeds were washed three times in distilled water, surface-disinfected with 75% alcohol, and germinated on wet paper in an incubator (16-h light/8-h dark photoperiod, 23–26°C). Every 10 seedlings were transplanted into a pot (12 cm in diameter; 15 cm in depth) and placed in the greenhouse for cultivation under a 16-h light/8-h dark photoperiod at approximately 23–26°C. The potting substrate consisted of sterilized soil (120 min, 121°C, 103 kPa) and vermiculite (3:1, V:V) to ensure drainage. Additionally, each pot was inoculated with 25 g of AMF mycorrhiza. An equal amount of sterilized soil was added to the non-inoculated treatment. Regular watering and seedling management were performed, and after 30 days of cultivation, the plants were watered with 100 mL of the corresponding NaCl concentration every 2 days. At 10 days later, the above-ground parts were sampled.

### 2.4 Experimental measurement methods

The mycorrhizal colonization rate of alfalfa roots after 40 days of growth was examined using the alkali dissociation-Trypan blue staining method (Jakobsen et al., 1992). Specifically, alfalfa roots were washed with distilled water, cut into segments of approximately 1.0 cm, then treated with 10% KOH, and boiled in a water bath at 90°C. After cooling, the roots were rinsed with distilled water, stained with Trypan blue dye in 90°C water bath, and then decolorized with lactate glycerin solution. The roots were randomly selected and placed on slides, and the mycorrhizal colonization rate was observed under a microscope. Five intact alfalfa plants with good growth were selected as a group to calculate mycorrhizal dependency using the formula (Hu et al., 2020).

The effects of AMF inoculation on alfalfa under salt treatment were determined by measuring various parameters. Fresh alfalfa height was measured from the root neck to the top of the plants, and fresh weight was recorded for the above-ground parts (Hu et al., 2020). Chlorophyll content was determined by acetone extraction, and the optical density (OD) of chlorophyll a (Chla) and chlorophyll b (Chlb) was measured at 663 and 645 nm, respectively. Total chlorophyll, Chla, and Chlb concentrations were calculated using the formula (Helaoui et al., 2020). The concentration of O_2_·^-^ was measured according to a previously described method (Liu et al., 2020). Malondialdehyde (MDA) content was estimated using the thiobarbituric acid (TBA) reaction (Ortega-Villasante et al., 2005), and pro content was determined using the methodology described by Bates et al. (1973). Physiological traits, including peroxidase (POD), SOD, and CAT activities, as well as soluble sugar (SS) and SP content, were measured using reagent kits (Suzhou Keming, Suzhou, China). Specific test procedures followed the manufacturer’s instructions for the reagent kits. Three technical replicates were performed for all index determinations.

### 2.5 Statistical analysis

Diversity and taxonomic analyses of microorganisms were performed using the I-Sanger cloud platform of Shanghai Meiji Biomedical Technology Co. Excel 2019 was used to summarize and organize the data, and all data were tested for normality and homogeneity of variance. Two-way ANOVA was used to analyze the interactive effects between G.m, G.e, and salt concentration. One-way ANOVA was performed to analyze the effects of the same salt concentration on indicators in the control group, G.m group, and G.e group, and Duncan multiple comparison (*P* < 0.05) was used to test their differences. IBM SPSS Statistics 27 was used for statistical analysis, and Origin 2021 was used for graphing.

## 3 Results

### 3.1 AMF identification

The effective sequences obtained by high-throughput sequencing were clustered into 156 OTUs. AMF species annotation was carried out by comparison with the MaarjAM database (https://www.maarjam.botany.ut.ee), and 40 species of six genera were identified (34 in *Glomus*, two in *Archaeospora*, one in *Diversispora*, one in *Gigaspora*, and two in *Paraglomus*), but no species of *Acaulospora* was detected (**Supplementary Table S1**).

**Figure 1:**
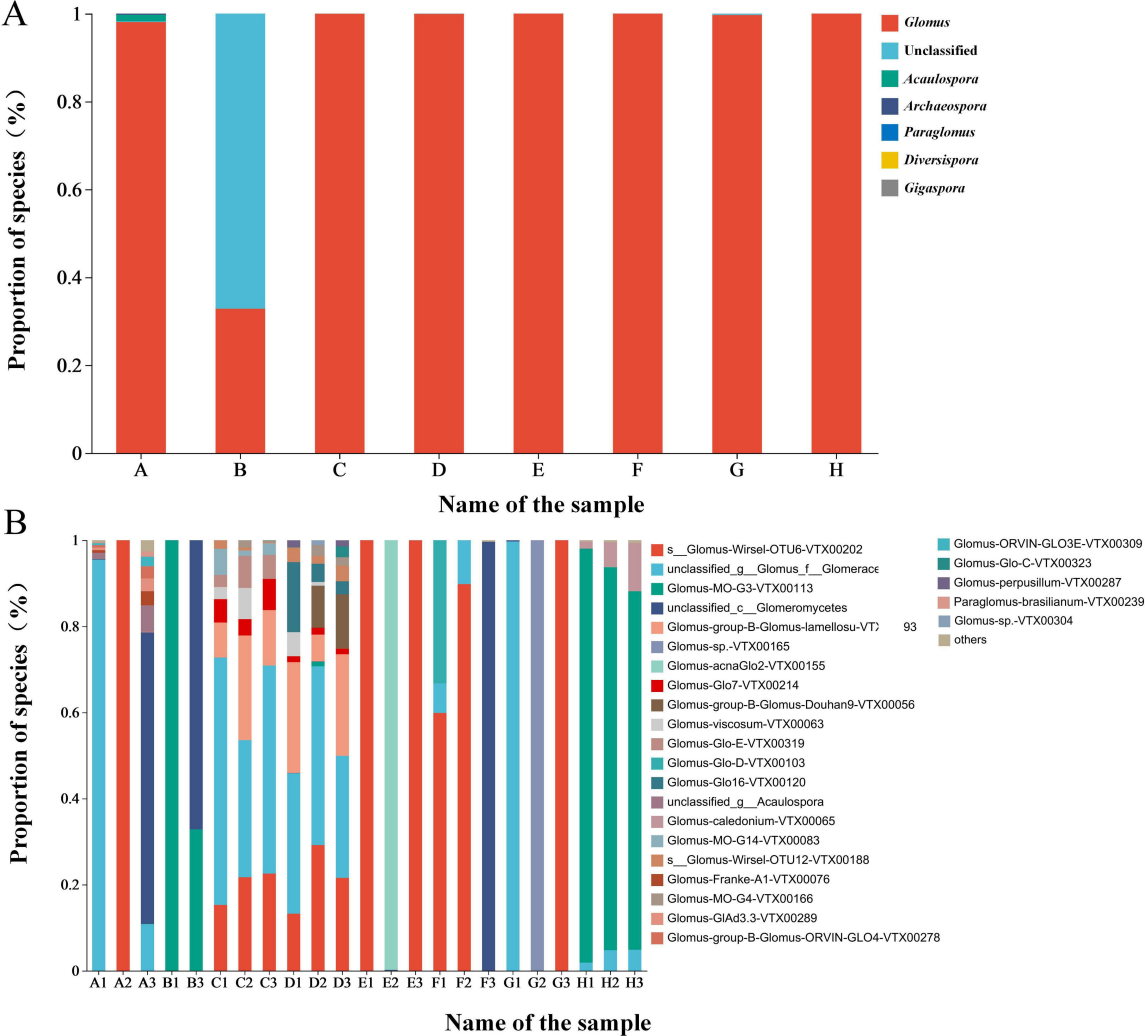
**(A)** Species composition of rhizosphere AMF of different plants at the genus level **(B)** Species composition of rhizosphere AMF of different plants at the species level.

At the genus level, *Glomus* was the dominant genus in the rhizosphere soils of plants, accounting for 89.33%, while unclassified accounted for 10.16% and was the second most abundant genus (**Figure 1A**). At the species level, *Glomus* Wirsel OTU6 VTX00202 had the highest relative abundance, accounting for 29.27%, making it the dominant species. This was followed by unclassified_g_Glomus_f_ Glomeraceae, for which no specific bacterial species was detected, and *Glomus* sp VTX00304 had the least relative abundance, accounting for only 0.05% (**Figure 1B**).

### 3.2 AMF diversity

The distribution of rhizosphere AMF varies among different plants. Among the eight soil samples, the most abundant AMF species were isolated from *Leymus chinensis*, *Tragopogon mongolicum*, and *Puccinellia tenuiflora*, with 12, 13, and 15 AMF species isolated, respectively. At least seven species of AMF were isolated from *Clematis hexapetala*. *Glomus mosseae* and *Glomus etunicatum* were the two most widely distributed AMF, being isolated from all soil samples (**Supplementary Table S2**).

The AMF community species richness and diversity indices varied significantly in the rhizosphere soils of different plants. The Sobs index indicated that species richness was highest in the *Arundinella anomala* samples (55.0 ± 22.0a), followed by *Taraxacum mongolicum* and *Puccinellia tenuiflora*, and lowest in the *Clematis hexapetala* samples (3.0 ± 2.0b). The Chao index showed that species richness was highest in the *Puccinellia tenuiflora* samples (45.33 ± 7.50a), followed by *Taraxacum mongolicum* and *Arundinella anomala*, and lowest in *Clematis hexapetala* (3.0 ± 2.0b). The Shannon and Simpson indices indicated similar results, showing a relatively high diversity in *Taraxacum mongolicum* and *Puccinellia tenuiflora* samples, while the lowest diversity was observed in the *Artemisia mongolica* sample (**Table 2**).

**Table 2:** Comparison of AMF community diversity index in the rhizosphere soil of different plants.

Number	Chao	Shannon	Simpson	Sobs
A	42.12 ± 7.47a	0.99 ± 0.57b	0.96 ± 0.07a	55.0 ± 22.0a
B	5.0 ± 2.0b	0.75 ± 0.06b	0.60 ± 0.18b	5.0 ± 2.0b
C	44.67 ± 3.79a	2.85 ± 0.20a	0.098 ± 0.007c	43.67 ± 2.08a
D	45.33 ± 7.50a	2.62 ± 0.14a	0.11 ± 0.01c	43.33 ± 4.04a
E	8.0 ± 4.0b	0.03 ± 0.008c	0.99 ± 0.01a	6.0 ± 2.65b
F	9.33 ± 3.21b	0.98 ± 0.12b	0.49 ± 0.11b	9.3 ± 3.21b
G	3.0 ± 2.0b	0.18 ± 0.03c	0.99 ± 0.003a	3.0 ± 2.0b
H	6.0 ± 0.58b	0.95 ± 0.09b	0.48 ± 0.01b	6.33 ± 0.58b

The values in the table are presented as mean ± standard deviation. Different lowercase letters indicate significant differences in diversity among different plant root systems and soil samples (*p*<0.05). Sobs is the actual observed value of species richness. The Chao index estimates the number of OTUs contained in a sample. The Shannon index estimates microbial diversity within a sample, while the Simpson index reflects community diversity, with larger values indicating lower community diversity.

### 3.3 Mycorrhizal colonization of alfalfa

**Figure 2:**
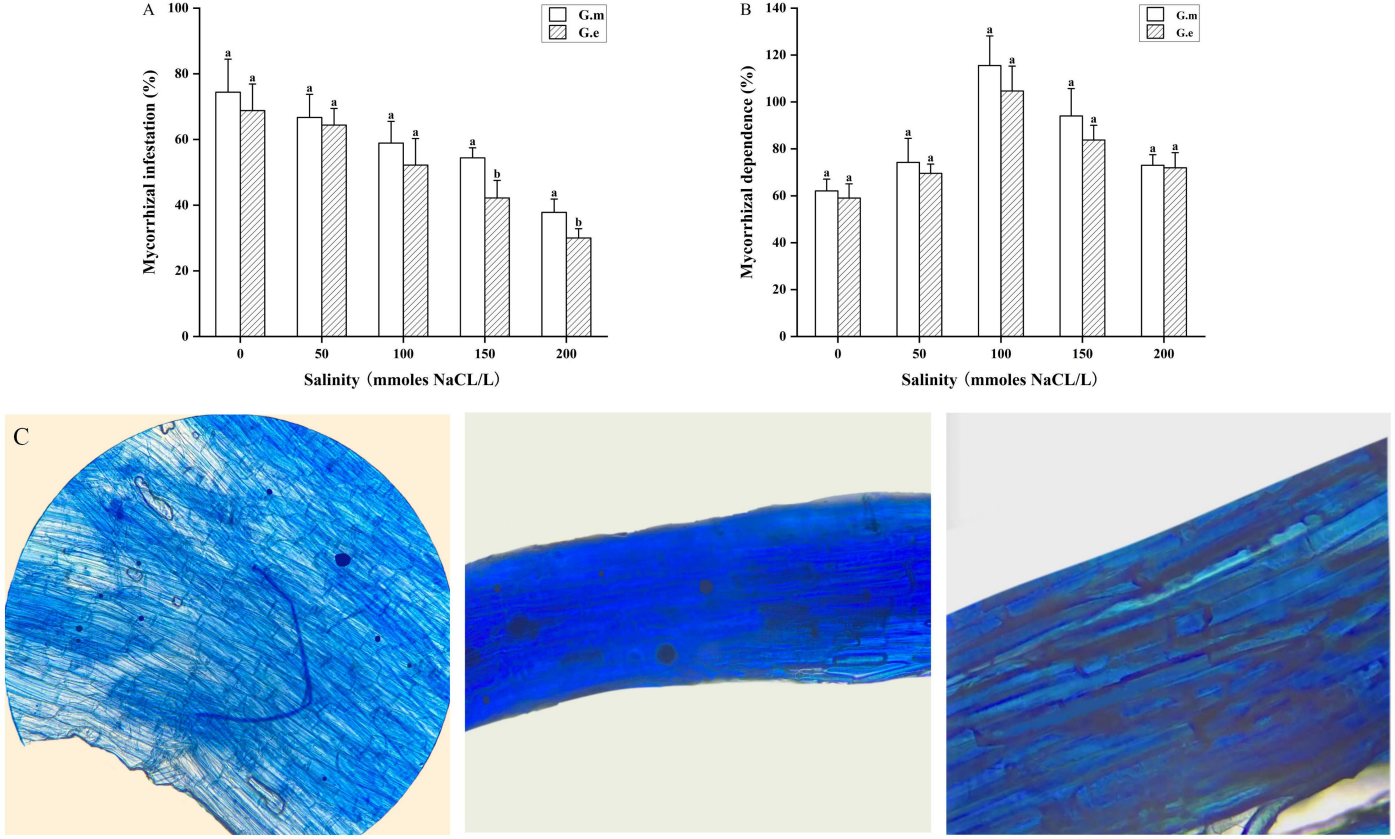
**(A)** Infestation rate of alfalfa under different treatments. **(B)** Dependence of alfalfa under different treatments. **(C)** Microstructure of AMF mycorrhiza of alfalfa. Bar groups with different lowercase letters indicate significant differences (P < 0.05) between treatments under the same salt concentration.

The native dominant AMF (G.m and G.e) inoculated with alfalfa under salt stress can infect and form a symbiotic system. Mycorrhizal colonization and dependence were higher in plants inoculated with G.m compared to those inoculated with G.e. Mycorrhizal colonization rates ranged from 33.3% to 76.6% for G.m and from 26.7% to 76.6% for G.e, while non-inoculated plants showed no colonization (**Figure 2C**). The colonization rate tended to decrease with increasing salt concentration. At 150 and 200 mM, inoculation with G.m resulted in significantly higher colonization rates than inoculation with G.e (*p* < 0.05, **Figure 2A**). Mycorrhizal dependence increased and then decreased with rising salt concentration, reaching a maximum at 100 mM (**Figure 2B**).

### 3.4 Interactive effect of AMF and salt concentration on indicators

Salt concentration had a highly significant impact on all indicators (*P* < 0.01, **Table 3**). Inoculation with G.m significantly affected all indicators except Chl b (*P* < 0.01). Inoculation with G.e did not affect Chl b, but it significantly impacted MDA (*P* < 0.05, **Table 3**). The interaction between inoculation with G.m, inoculation with G.e, and salt concentration had a highly significant impact on Chl b, Chl, Pro content, and O_2_·^-^ production rate (*P* < 0.01, **Table 3**) but no significant impact on plant height, fresh weight, SS, SP, and POD.

**Table 3:** Two-way ANOVA of the effects of G.m, G.e, and salt concentration on indicators.

Indicators	G.m	Salt concentration	G.m * salt concentration	G.e	Salt concentration	G.e * salt concentration
P H	55.711**	49.681**	1.102	47.779**	67.853**	1.971
FW	31.642**	39.389**	1.475	14.433**	54.655**	1.385
Chla	21.905**	223.856**	4.341*	23.398**	292.579**	3.931*
Chlb	3.924	246.898**	14.538**	0.815	336.614**	11.002**
Chl	28.724**	573.82**	17.453**	17.825**	1159.289**	16.579**
MDA	40.082**	77.036**	4.598**	7.753*	77.621**	1.235
Pro	298.497**	180.912**	52.574**	180.149**	165.831**	37.395**
SP	142.61**	170.118**	0.534	85.788**	426.143**	1.547
SS	24.725**	18.926**	1.763	15.239**	15.249**	0.279
O_2_·^-^	230.825**	226.896**	22.259**	78.219**	217.43**	5.616**
SOD	124.855**	38.398**	3.28*	87.396**	43.431**	2.468
POD	539.642**	185.224**	0.932	555.219**	254.48**	1.654
CAT	106.515**	148.549**	4.01*	89.514**	160.855**	1.749

The figures denote F-values. * denotes significant difference at *p*<0.05. ** denotes significant difference at *p*<0.01. P H indicates plant height in the table.

### 3.5 Effects on alfalfa growth indicators

**Figure 3:**
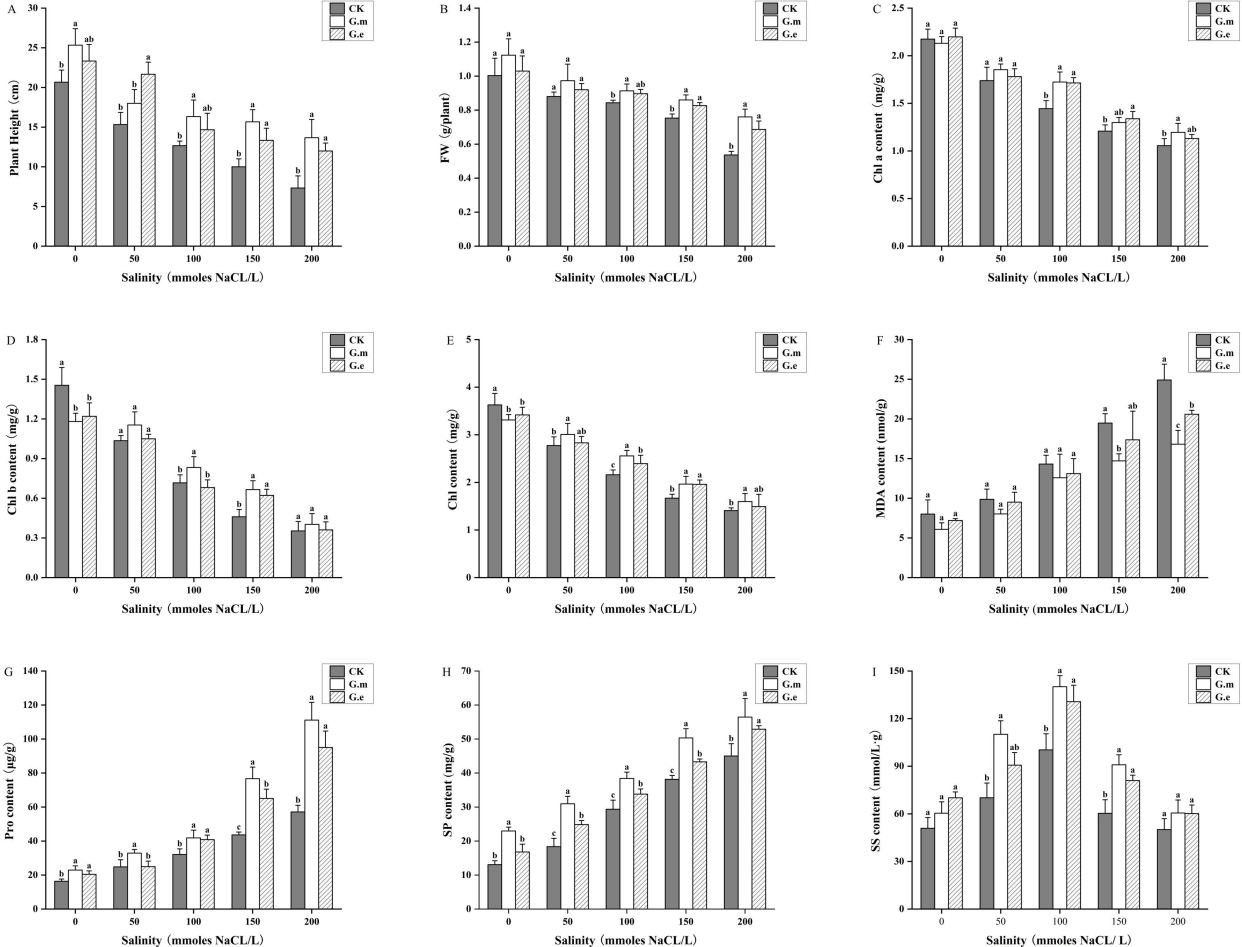
**(A)** Alfalfa plant height under different treatments. **(B)** Fresh weight of alfalfa under different treatments. **(C)** Chl a content of alfalfa under different treatments. **(D)** Chl b content of alfalfa under different treatments. **(E)** Chlorophyll content of alfalfa under different treatments. **(F)** MDA content in alfalfa under different treatments. **(G)** Pro content of alfalfa under different treatments. **(H)** SP content of alfalfa under different treatments. **(I)** SS content of alfalfa under different treatments. Bar groups with different lowercase letters indicate significant differences (P < 0.05) between treatments under the same salt concentration. Data are means ± standard error.

The plant height and fresh weight of alfalfa decreased with increasing salt concentration. AMF-inoculated plants at corresponding salinity levels were significantly taller and had higher fresh weight than control plants (*P* < 0.05). The plant height and fresh weight of AMF-inoculated plants were significantly different from control plants at 150 and 200 mM (*P* < 0.05, **Figures 3A, B**). Plant height increased by 56.67% and 33.33% and by 86.36% and 63.63%, while fresh weight increased by 14.16% and 9.7% and by 41.61% and 27.95%, respectively, with increasing salt concentration. There was no significant difference between G.m and G.e.

### 3.6 Effects on physiological indicators of alfalfa

Chl a, Chl b, and total chlorophyll content decreased with increasing salt concentration. Chl a content in alfalfa inoculated with G.m and G.e was significantly higher compared to non-inoculated plants at 100 mM (*P* < 0.05, **Figure 3C**). The trends for Chl b and total chlorophyll content were similar, with AMF inoculation showing higher levels than non-inoculated plants without salt stress. The Chl b and total chlorophyll contents in G.m- and G.e-inoculated plants were dramatically higher than in control plants at 100 and 150 mM (*P* < 0.05, **Figures 3D, E**). The chlorophyll content in G.m-inoculated plants was significantly higher than in non-inoculated plants at 200 mM. MDA content increased with increasing salt concentrations. AMF inoculation resulted in lower MDA content compared to control plants at the same salt concentration, reaching a maximum at 200 mM, which was significantly different from control plants (*P* < 0.05, **Figure 3F**).

### 3.7 Effects on osmoregulatory substances of alfalfa

The trends for Pro and SP content were similar, increasing with rising salt concentrations. AMF-inoculated plants at the same salt concentrations had significantly higher levels than control plants (*P* < 0.05). The Pro content in alfalfa inoculated with both G.m and G.e reached the highest levels at 200 mM, with G.m showing the most significant increase from 43.13 to 111.12 μg/g (*P* < 0.05, **Figure 3G**). The SP content in plants inoculated with G.m and G.e was significantly higher at 50, 100, and 150 mM (*P* < 0.05), but the difference disappeared at 200 mM (*P* < 0.05, **Figure 3H**). SS content in alfalfa increased and then decreased with rising salt concentrations, peaking at 100 mM, where the difference between AMF-inoculated and control plants was significant (*P* < 0.05). No significant difference was observed at 200 mM (**Figure 3I**).

### 3.8 Effects on antioxidant enzyme activities of alfalfa

**Figure 4:**
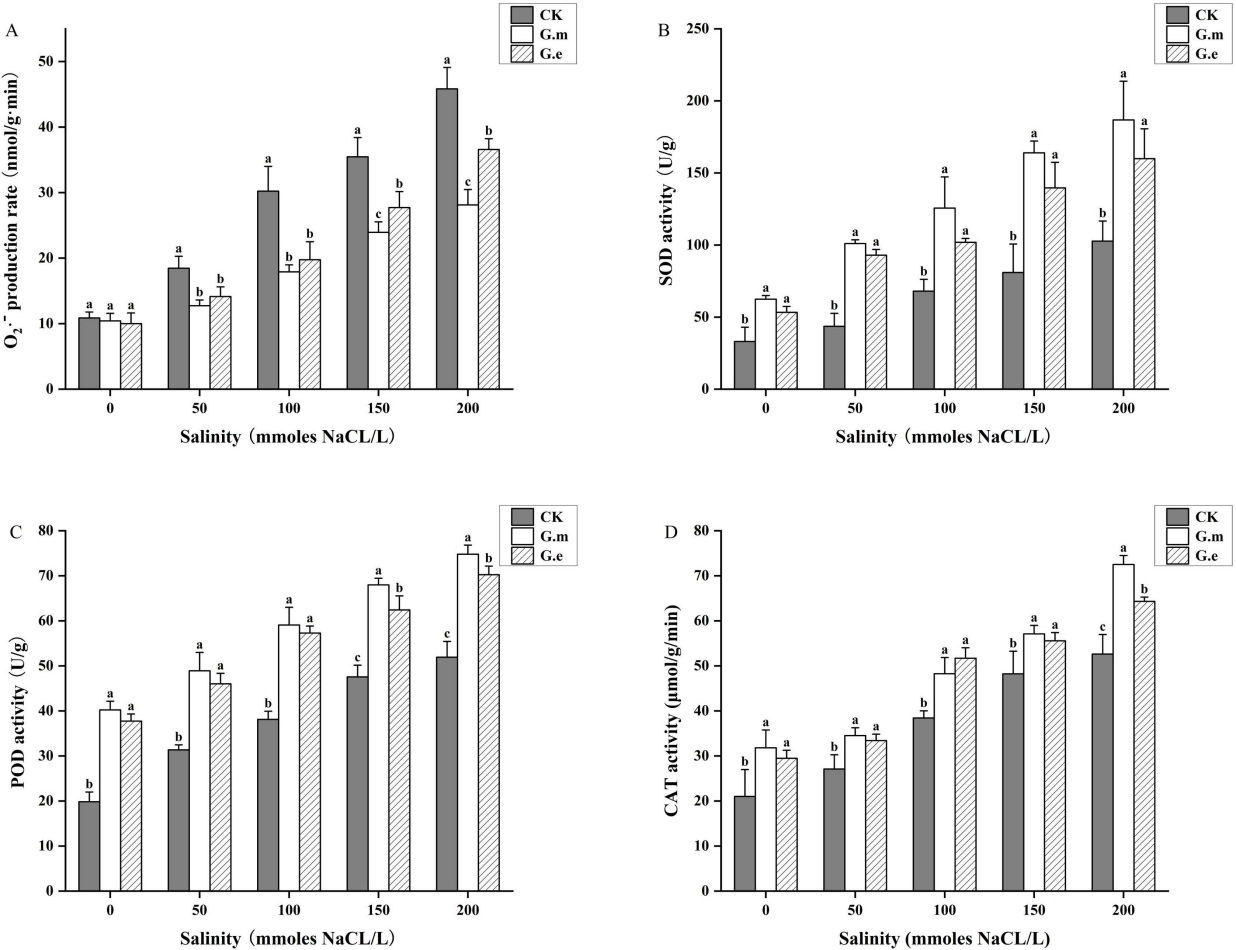
**(A)** O2·- production rate of alfalfa under different treatments. **(B)** SOD activity of alfalfa under different treatments. **(C)** POD activity of alfalfa under different treatments. **(D)** CAT activity of alfalfa under different treatments. Bar groups with different lowercase letters indicate significant differences (P < 0.05) between treatments under the same salt concentration. Data are means ± standard error.

The O_2_·^-^ production rate in alfalfa under salinity stress increased with NaCl concentration. There was no difference between AMF-inoculated and non-inoculated plants without salt stress. However, AMF-inoculated plants showed significantly lower O_2_·^-^ production rates than non-inoculated plants with increasing salt concentration (*P* < 0.05, **Figure 4A**). Salt stress and AMF inoculation significantly impacted antioxidant enzyme activity. SOD, POD, and CAT activities showed similar trends, increasing with rising salt concentrations. The activity levels in AMF-inoculated plants were significantly higher compared to control plants (*P* < 0.05), with the maximum observed at 200 mM. SOD activity in plants inoculated with G.m and G.e increased by 131.69% and 113.10%, respectively, compared to the control at 50 mM (*P* < 0.05, **Figure 4B**). POD and CAT activities in plants inoculated with G.m were significantly higher than in those inoculated with G.e at high concentrations (*P* < 0.05, **Figures 4C, D**).

### 3.9 Correlation coefficients between physiological indicators of alfalfa

Pearson correlation analysis of 12 physiological indicators of alfalfa inoculated with AMF under salt stress revealed several significant relationships. The Pro content was significantly positively correlated with plant height and MDA content. The SS content was highly obviously positively correlated with the Chl b content and significantly positively correlated with the Chl content. The SP content showed a significant positive correlation with SOD and POD activities. CAT activity was negatively correlated with Chl content, while SS content had a positive correlation with POD activity (**Table 4**).

**Table 4:** Correlation coefficient of the physiological indicators of AMF-inoculated alfalfa under salt stress.

Indicator	PH	FW	Chl a	Chl b	Chl	SS	SP	Pro	MDA	SOD	POD	CAT
PH	1											
FW	-0.353	1										
Chl a	0.132	0.217	1									
Chl b	0.414	0.213	0.390	1								
Chl	0.364	0.230	0.797**	0.863**	1							
SS	0.010	0.449	0.322	0.792**	0.701*	1						
SP	-0.333	0.354	-0.552	-0.317	-0.499	-0.055	1					
Pro	0.882**	-0.382	0.256	0.400	0.418	0.037	-0.282	1				
MDA	-0.596	-0.031	0.061	-0.213	-0.104	0.047	-0.308	-0.756*	1			
SOD	-0.010	0.487	-0.300	0.287	0.052	0.566	0.664*	-0.131	-0.162	1		
POD	-0.539	0.259	-0.557	-0.717	-0.799**	-0.564	0.638*	-0.495	-0.070	0.002	1	
CAT	-0.240	0.139	-0.324	-0.786	-0.709*	-0.713*	0.490	-0.144	-0.300	-0.173	0.847**	1

* denotes significant difference at *p*<0.05. ** denotes significant difference at *p*<0.01.

## 4 Discussion

### 4.1 AMF diversity of salinity-tolerant plants of Songnen grassland

AMF, as a type of “biofertilizer,” not only promotes the absorption of mineral elements and water in plants but also improves resistance to diseases and adverse conditions, which is crucial for agroforestry production (Marro et al., 2022). We analyzed the distribution and diversity of rhizosphere AMF in the Songnen saline-alkali grassland ecosystem in China. Different host plants have specific functions, physiological metabolisms, and root exudates, leading to varied AMF community diversity among different vegetation types (Kokkoris et al., 2020). AMF community also vary with soil conditions, nutritional status, and microbial group dynamics (Frew, 2019; Blažková et al., 2021), as different AMF have distinct nutrient acquisition capabilities (Zhang et al., 2021). A previous study showed that the soil nutrient levels on the eastern slope of the Helan Mountains first increased and then decreased with increasing altitude, and the diversity of soil AMF communities followed the same trend (Shao et al., 2024). Geographical distance and climatic conditions can also have indirect effects (Li et al., 2023b). In our study, 40 AMF species from six genera were identified from the rhizosphere soil using high-throughput sequencing, with *Glomus* detected as the dominant genus. Our results align with the dominant genera of rhizosphere AMF in *Inula japonica* and *Iris lactea* in the Songnen saline-alkali grassland. Among them, *G. mosseae* and *G. etunicatum* were isolated from all soil samples and were the most widely distributed AMF, indicating optimal affinity with the experimental plants. Furthermore, *Glomus* has been identified as a dominant genus in many studies on Songnen saline-alkali grasslands and in various ecosystems (Shao et al., 2024; Guo et al., 2022; Zheng et al., 2022). This phenomenon could be related to the spore formation pattern of *Glomus* and the suitable concentration of organic matter and N and P elements in the soil, improving its competitiveness (Avio et al., 2006). Different rhizosphere AMF species were observed in the rhizospheres of different plants, showing that different host plants affect the AMF distribution (Sýkorová et al., 2007).

The diversity and structure of soil microbial communities are crucial for soil function and the ecological environment (Lin et al., 2022). Research has shown that the diversity indices of rhizosphere AMF in different plant soils, such as Sobs, Chao, Shannon, and Simpson, vary significantly. Consistent with previous studies, plant communities greatly affect the AMF community diversity (Brigido et al., 2017). AMF and host plants exhibit a certain preference when forming symbiotic systems, and their affinity and mutual selectivity determine the survival and development of AMF to a significant extent (Croll et al., 2008; Wang et al., 2024a). This might be associated with the different nutritional requirements and types of root cells in various plants. Studies have found that differences in longitude, latitude, or altitude create varying water, light, temperature, and soil gradients, affecting the AMF diversity (Zhao et al., 2020). Furthermore, abiotic factors such as insect herbivores, climate change, N and P addition, and human interference influence AMF diversity (Mei et al., 2022b; Li et al., 2023b). A variety of rhizosphere AMF symbionts are distributed in the Songnen saline land, allowing for the selection and utilization of the optimal AMF. This provides a crucial theoretical basis for restoring saline-alkali land.

### 4.2 Effect of dominant AMF on alfalfa colonization rate

AMF can form mutualistic symbioses with plant roots, acting as a symbiotic rhizosphere barrier to decrease the harmful impact of salinity stress (Malik et al., 2022). The higher the mycorrhizal colonization rate, the stronger the symbiotic ability between plants and AMF. In this study, AMF inoculation on alfalfa resulted in a decrease in mycorrhizal colonization rate with increasing NaCl concentration. This indicates that factors affecting AMF colonization status are not only related to the affinity between plants and AMF but also to environmental factors, especially soil conditions that affect plant root growth (Rúa et al., 2016; Liu et al., 2023b). Research suggests that salt stress impacts mycorrhizal colonization by inhibiting spore germination and mycelial growth (Zhang et al., 2024b). There is a degree of mutual selectivity between AMF and host plants, and the AMF selected by plants may vary in different environments. The study showed that inoculation with *G. mosseae* resulted in a higher colonization rate than *G. etunicatum* under different NaCl stress levels. Plant AMF mycorrhizal dependence is an indicator of the symbiotic relationship between plants and AMF. In this experiment, mycorrhizal dependence showed an increasing and then decreasing trend with rising salt concentration, similar to the findings in maize (Ahmed et al., 2023). This may be because alfalfa can tolerate low salt concentrations, and AMF gradually plays a role as the salt concentration increases to 100 mmol/L. However, at 150 mmol/L, the growth of alfalfa is disrupted, inhibiting the elongation of AMF mycelium and reducing alfalfa’s mycorrhizal dependence. Additionally, reduced mycorrhizal dependence may also be due to the inhibition of AMF development and structure by salinity stress (Hashem et al., 2019).

### 4.3 Effects of dominant AMF on alfalfa

Plant growth and development are severely restricted by salinity stress (Guo et al., 2024). Salt stress is closely related to water status, increasing ion content and reducing the water absorption capacity of plants, leading to a rapid decrease in biomass (Li et al., 2019). Our study showed that the above-ground height and fresh weight of alfalfa decreased with increasing salt concentration. However, inoculation with AMF effectively improved plant height and fresh weight under certain salt stress conditions. This demonstrates that AMF inoculation can effectively promote the growth and development of alfalfa. AMF colonization can protect the host plant from the harmful impacts of salt stress (Santander et al., 2019). Our results agree with the findings that AMF colonization in wheat increased shoot length and fresh weight and that AMF inoculation increased the shoot biomass of tomato under salt treatment (Huang et al., 2023; Liu et al., 2023a). Inoculation with AMF under salt stress may expand the root absorption area, making it easier for plants to absorb water and nutrients, thus promoting growth (Wang et al., 2022).

Changes in chlorophyll content can reflect the impact of salinity stress on plant photosynthesis. In this study, increasing salt concentration had a significant negative impact on chlorophyll content in alfalfa leaves, consistent with previous studies (Hashem et al., 2018; Huang et al., 2023). The likely reason is that high salt concentrations affect the role of Mg in the plant, slowing down the chlorophyll synthesis rate. Chl a, Chl b, and total chlorophyll content in alfalfa inoculated with AMF at higher NaCl concentrations (100 and 150 mM) showed significant differences from control plants. Similar results have been observed in studies on tomato (Kong et al., 2020), indica rice (Tisarum et al., 2020), and *Citrus aurantium* (Hadian-Deljou et al., 2020). Notably, the differences reduced at 200 mM, with inoculation with *G. mosseae* being more effective than *G. etunicatum*. The findings indicated that the presence of AMF promoted chlorophyll synthesis within a certain range. This promotion may be due to AMF’s contribution to maintaining the intact ultrastructure of chloroplast thylakoids and mitochondria (Liu et al., 2023c) and upregulating the expression and activity of enzymes related to chlorophyll synthesis (Li et al., 2023a). Additionally, the increase in chlorophyll content in mycorrhizal plants may be due to the release of cytokinin-like substances by the fungus, promoting chloroplast development (Navarro et al., 2014).

Plants subjected to salt stress mobilize osmoregulatory mechanisms, produce osmoregulatory substances, and balance cell membrane permeability to resist injury (Xu et al., 2023). Our study found that SS, SP, and Pro content increased with rising salt concentrations and were significantly higher in AMF-inoculated plants compared to non-inoculated ones. This suggests that plants resist salinity stress by accumulating SS, SP, and Pro and that AMF can effectively alleviate the harmful effects of NaCl stress on the osmotic metabolic system. Similar results were observed in studies on poplar seedlings and *Xanthoceras sorbifolium* Bunge (Zong et al., 2023; Han et al., 2024). This indicates that AMF inoculation may increase the secretion of osmoregulatory substances under salinity stress, reduce osmotic potential, maintain normal cell metabolism, and improve plant salt resistance (Zong et al., 2023). Notably, there was no significant difference in SS content between AMF-inoculated and uninoculated plants at 200 mM, suggesting that excessively high salt concentrations may impair plant metabolic activity, reducing SS synthesis and accelerating decomposition.

As salt concentration increases, the plant’s antioxidant system fails to clear all reactive oxygen species (ROS), leading to membrane lipid peroxidation and increased production of MDA (Latef and Chaoxing, 2011; Ali et al., 2019). In this study, the MDA content in alfalfa increased with higher salt concentrations and was significantly reduced by AMF inoculation. This is consistent with the research of Wang et al., which showed that AMF inoculation in maize under salt stress reduced the MDA content (Wang et al., 2020). Increased MDA may be associated with the increase in antioxidant enzyme activities caused by oxidative damage (Wang et al., 2023). Salinity and other stressors can induce the formation of ROS, with O_2_·^-^ being a particularly toxic type that is highly reactive and harmful, causing damage to proteins and lipids (Chen et al., 2020). AMF inoculation significantly reduced the O_2_·^-^ production rate under salt stress compared to non-inoculated plants, indicating that AMF inoculation could reduce the degree of membrane lipid peroxidation in alfalfa.

Salt stress can cause lipid peroxidation, carbohydrate oxidation, and enzyme activity damage in cell membranes, leading to the production of excessive ROS that damage cells (Yan et al., 2021). Antioxidant enzymes such as SOD, POD, and CAT are commonly found in plants and help reduce damage to plant membrane systems under adverse conditions. AMF contribute to the direct or indirect clearance of ROS and increase antioxidant enzyme activity in plants under salinity stress (Mehta and Vyas, 2023), which is consistent with our results. We observed that SOD, POD, and CAT activities in alfalfa increased with rising salt concentrations and were significantly higher in AMF-inoculated plants compared to non-inoculated ones. Research has shown that AMF inoculation significantly increases antioxidant oxidase activities in *Gossypium hirsutum* and *Cucumis sativus* L. under salinity stress (Hashem et al., 2018; Zhang et al., 2024b). It can be inferred that AMF colonization benefits the synthesis of antioxidant enzymes and improves the defense system of antioxidant enzymes (Evelin et al., 2019). Moreover, the higher activity of antioxidant enzymes in AMF mycorrhizal plants could be related to the lower accumulation of lipid peroxidation (Latef and Chaoxing, 2011), resulting in reduced oxidative damage.

## 5 Conclusion

The diversity of AMF in the rhizospheres of eight common plants in the Songnen saline-alkali grassland and the impact of dominant native AMF species on the salt tolerance of alfalfa are explored for the first time. A total of 40 species of AMF from six genera were identified from rhizosphere soil samples of different plants in Songnen grassland. G.m and G.e were identified as dominant AMF species. The utility of these two dominant AMF species from the locations in alleviating salinity stress in pot-cultured alfalfa was tested in greenhouse conditions. The significant findings were that AMF improve the salt-alkali tolerance of alfalfa under salt stress by increasing the plant height, fresh weight, chlorophyll, SS, SP, Pro content, and SOD, POD, and CAT activities while reducing the MDA content. Future research should focus on multiple bacterial strains to improve alfalfa resistance, especially on cultivating and propagating multiple native bacterial strains, to provide a theoretical basis for the improvement of saline-alkali land.

## Data Availability

The raw data supporting the conclusions of this article will be made available by the authors, without undue reservation.
