# Supplementary material for: Arbuscular mycorrhizal fungi communities and promoting the growth of alfalfa in saline ecosystems of northern China
Source: Front Plant Sci. 2024 Aug 29;15:1438771. doi: 10.3389/fpls.2024.1438771 (PMC11390447; doi:10.3389/fpls.2024.1438771)
Supplement: Supplementary file 1 [file DataSheet1.docx]

Table S1 Virtual taxa of rhizosphere AMF of different plants with high-throughput sequencing

| Genus | Virtual taxa | |
| --- | --- | --- |
| *Glomus* | *Glomus* Franke A1 VTX00076  *Glomus* Franke A1 VTX00269  *Glomus* Glo C VTX00323  *Glomus* Glo D VTX00103  *Glomus* Glo E VTX00319  *Glomus* Glo7 VTX00214  *Glomus* Glo16 VTX00120  *Glomus* PSAMG1 VTX00291  *Glomus* Yamato2005 D VTX00084  *Glomus* Yamato2005 D VTX00224  *Glomus* sp. VTX00165  *Glomus* sp. VTX00279  *Glomus* sp. VTX00304  *Glomus* caledonium VTX00065  *Glomus* Douhan9 VTX00056  *Glomus* lamellosu VTX00193  *Glomus* perpusillum VTX00287 | *Glomus* GlAc3.1 VTX00190  *Glomus* GlAd3.3 VTX00289  *Glomus* MO G3 VTX00113  *Glomus* MO G4 VTX00166  *Glomus* MO G13 VTX00115  *Glomus* MO G14 VTX00083  *Glomus* MO G16 VTX00072  *Glomus* MO G22 VTX00125  *Glomus* Wirsel OTU12 VTX00188  *Glomus* Wirsel OTU6 VTX00202  *Glomus* acnaGlo2 VTX00155  *Glomus* acnaGlo7 VTX00057  *Glomus* viscosum VTX00063  *Glomus* ORVIN GLO3B VTX00223  *Glomus* ORVIN GLO3E VTX00309  *Glomus* ORVIN GLO3D VTX00310  *Glomus* ORVIN GLO4 VTX00278 |
| *Archaeospora* | *Archaeospora* Other1 VTX00005 | *Archaeospora* sp. VTX00009 |
| *Paraglomus* | *Paraglomus* brasilianum VTX00239 | *Paraglomus* occultum VTX00238 |
| *Diversispora* | *Diversispora* MO GC1 VTX00060 |  |
| *Gigaspora* | *Gigaspora* decipiens VTX00039 |  |

Note: The values in the table are the names and virtual species numbers of different plant rhizosphere AMF genera identified by high-throughput sequencing

Table S2 Distribution of rhizosphere AMF of different plants in grassland

| Genus | Specie | A | B | C | D | E | F | G | H |
| --- | --- | --- | --- | --- | --- | --- | --- | --- | --- |
| *Glomus* | *G. deserticola* |  |  | ＋ |  |  |  |  |  |
|  | *G. mosseae* | ＋ | ＋ | ＋ | ＋ | ＋ | ＋ | ＋ | ＋ |
|  | *G. pansihalos* |  |  | ＋ | ＋ | ＋ |  |  | ＋ |
|  | *G. convolutum* | ＋ |  | ＋ | ＋ |  | ＋ | ＋ |  |
|  | *G. magnicaule* |  | ＋ | ＋ | ＋ |  |  |  | ＋ |
|  | *G. etunicatum* | ＋ | ＋ | ＋ | ＋ | ＋ | ＋ | ＋ | ＋ |
|  | *G.tenebrosum* |  | ＋ |  |  | ＋ | ＋ |  | ＋ |
|  | *Glomus sp.1* |  |  | ＋ | ＋ |  | ＋ |  | ＋ |
| *Acaulospora* | *A. foveata* |  | ＋ |  | ＋ |  | ＋ | ＋ |  |
|  | *A. laevis* | ＋ | ＋ | ＋ | ＋ |  | ＋ |  |  |
|  | *A. lacunosa* |  |  | ＋ | ＋ |  | ＋ |  | ＋ |
|  | *A. mellea* | ＋ |  |  | ＋ | ＋ |  | ＋ |  |
|  | *A.rehmii* | ＋ |  |  |  | ＋ |  |  | ＋ |
| *Rhizophagus* | *R. fasciculatus* |  | ＋ | ＋ | ＋ | ＋ |  |  |  |
|  | *R. manihotis* |  | ＋ |  | ＋ |  |  |  |  |
|  | *R. intraradices* | ＋ | ＋ | ＋ | ＋ | ＋ |  | ＋ |  |
|  | *R. cluram* | ＋ |  |  | ＋ |  | ＋ |  |  |
| *Ambispora* | *A. leptoticha* |  | ＋ | ＋ |  |  |  |  | ＋ |
|  | *A.gerdemannii* | ＋ |  | ＋ |  |  | ＋ |  |  |
| *Claroideoglomus* | *C. claroideum* | ＋ |  |  |  | ＋ |  | ＋ |  |
| *Gigaspora* | *Gi. decipiens* |  | ＋ |  |  | ＋ |  |  | ＋ |
| *Funneliformis* | *F. constrictum* |  | ＋ |  | ＋ |  | ＋ |  |  |


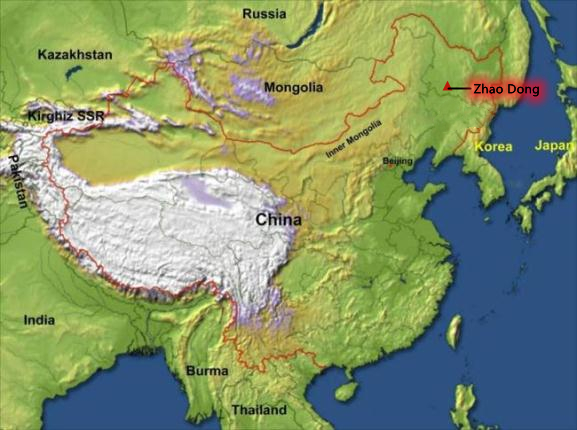


Figure S1 Location map of the study area in China


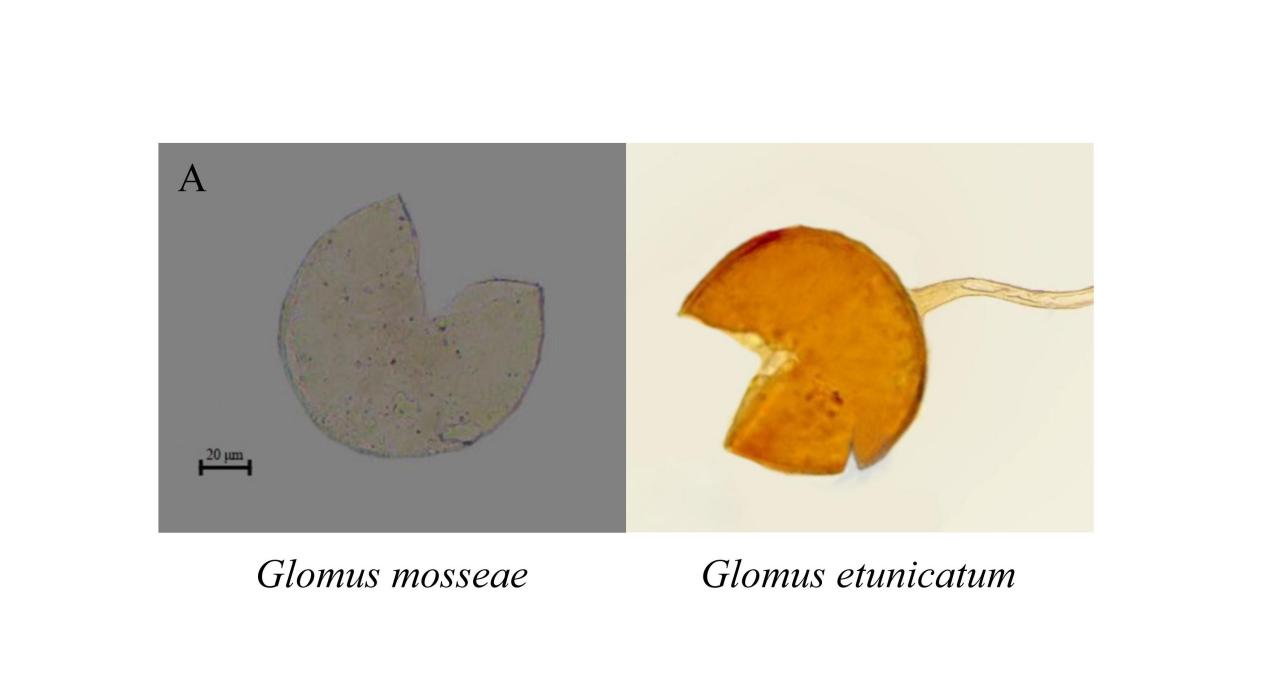


Figure S2 Dominant AMF spores in rhizosphere soil of different plants in Songnen grassland
